# Dams and Disease Triggers on the Lower Mekong River

**DOI:** 10.1371/journal.pntd.0002166

**Published:** 2013-06-13

**Authors:** Alan D. Ziegler, Trevor N. Petney, Carl Grundy-Warr, Ross H. Andrews, Ian G. Baird, Robert J. Wasson, Paiboon Sithithaworn

**Affiliations:** 1 Geography Department, National University of Singapore, Singapore; 2 Department of Ecology and Parasitology, Zoology Institute, Karlsruhe Institute of Technology, Karlsruhe, Germany; 3 Department of Parasitology, Liver Fluke and Cholangiocarcinoma Research Center, Medical Faculty, Khon Kaen University, Khon Kaen, Thailand; 4 Faculty of Medicine, Imperial College London, United Kingdom; 5 Department of Geography, University of Wisconsin, Madison, Wisconsin, United States of America; National Institute of Parasitic Diseases, China CDC, China

Ongoing and proposed construction of several large hydropower dams along the mainstream Mekong River and various tributaries has created a number of unanswered environmental and societal questions for governments and communities in Cambodia, China, Lao PDR, Myanmar, Thailand, and Vietnam [1]–[3]. Most concern over the controversial dam-building projects focuses on the extent to which river health and food security will be affected negatively. Foremost, the 85 or more proposed dams threaten to reduce the diversity and abundance of freshwater fish, the major animal protein source for many of the 67 million inhabitants of the Mekong River basin [4]–[7].

**Figure 1:**
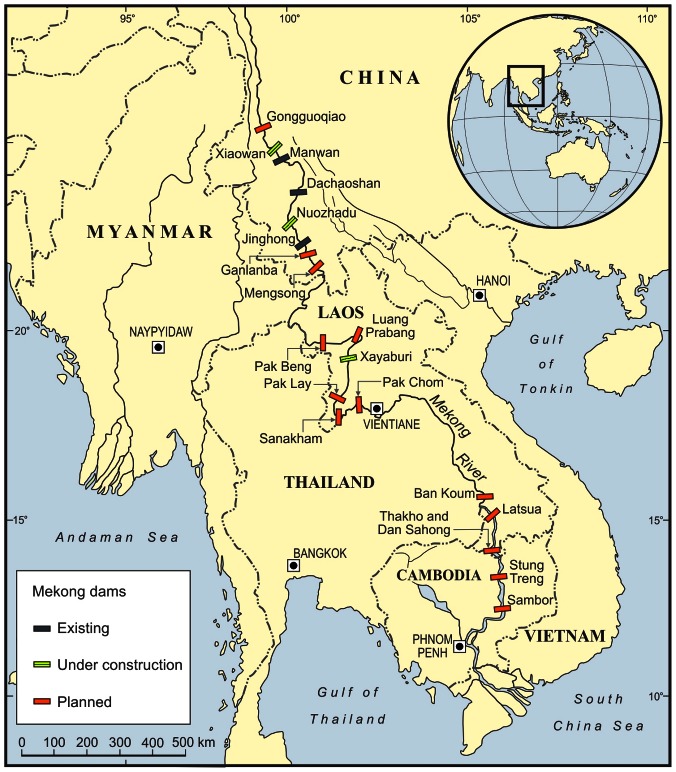
Proposed and existing dams on the man stem of the Mekong river. More than 85 dams are now proposed to be built on the main channel and tributaries of the Mekong River in Southeast Asia (only those on the main stem are shown). The Xayaburi Dam is the first of several dams proposed for the Lower Mekong mainstream. Three dams are already built on the main branch of the upper Mekong River in China (Manwan, Dachaoshan, Jinghong); two more are under construction (Xiaowan, Nuozhadu) and three more are planned.

The Xayaburi Dam is the first of 12 dams proposed for the Lower Mekong River (Figure 1). Delayed in mid-2012 in response to international and regional pressure to reevaluate potential environmental consequences, the project has recently been approved again. It is projected to affect the livelihoods of hundreds of thousands of people through detrimental impacts on river ecology—fishing in particular. This is just one dam. The impact of the entire network could be much more devastating. A recent study simulated catastrophic losses to fish productivity and diversity as a result of construction of 85 dams on the main channel and tributaries [7].

From a hydro-geomorphological standpoint, the cascade of dams would fundamentally change the river flow regime that maintains fish habitat and sediment transport. Not only would seasonal flows be affected, the magnitude and timing of high-flow events would lessen, greatly affecting aquatic environments [8]. Seasonally inundated floodplains adjacent to the river channels are productive feeding, spawning, and nursing habitats for important Mekong fishes. Perhaps more important, deep pools in some sections of the main channel serve as fish refuge habitats during the dry season [9]. Furthermore, sediments that are naturally transported in the river are vital to maintaining aquatic habitats such as pools and sand bars in river channels, as well as downstream deltas. Aquatic life also depends on sediment-associated nutrients, detritus, and organic debris.

Habitat destruction caused by a lack of flushing during large flood events and reductions of sediment could result in a loss of fish diversity and abundance, further adding to impacts caused by disruption of migration patterns. The implementation of fish ladders or other fish passage systems—such as those proposed for the Xayaburi dam—are anticipated to have a limited effect for Mekong river fish [3]. Fish would still have to navigate through the turbines to pass downstream. Furthermore, fish passes would provide no relief for habitat loss, nor would they allow the downstream drift of fish larvae.

**Figure 2:**
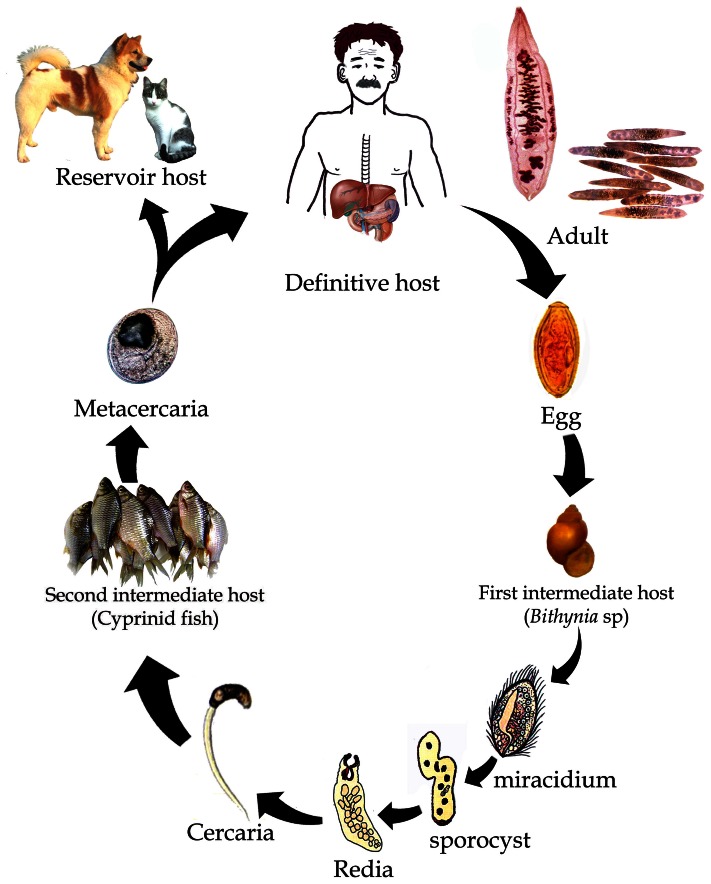
The life cycle of the *O. viverrini* parasite. The life cycle of the *O. viverrini* parasite involves intermediate snail (*Bithynia* sp.) and native fish (cyprinid sp.) hosts, which are commonly found in wetland environments within the Mekong Basin, as well as in definitive human and carnivore reservoir hosts. Alarmingly, *O. viverrini* currently infects more than 10 million people in Thailand and Laos PDR [13]. The cholangiocarcinoma caused by the *O. viverrini* liver fluke will likely kill hundreds of thousands of people in the coming decades [14]. The wide variability of incidence across the region is largely related to culture and eating behavior [12]. There are also insufficient data to make accurate assessments in countries such as Laos and Cambodia. Humans become infected by ingesting metacercariae in uncooked fish [14]. The ingested metacercariae excyst in the duodenum and enter the bile duct, where they develop into sexually mature adult worms. The eggs produced by the adults are discharged with bile fluid into the intestine, and out of the body with the feces. When viable eggs from improperly treated waste reach a body of freshwater and are ingested by an appropriate snail, miracidia hatch and develop into sporocysts and rediae. The rediae gave rise to free-swimming cercariae and, when exposed to appropriate cyprinid species of fish (the second intermediate hosts), the cercariae penetrate into the tissues or skin of freshwater fish and become fully infective metacercariae. When humans then eat these fish raw or undercooked, the life cycle is completed.

While the linkage between dam building, fish ecology, and food security is obvious, limited attention has been given to the potential threat Mekong dams will pose to public health via disease ecology and food safety [10]. The rapidly changing Mekong River basin harbors a wide diversity of water- and food-associated pathogens. These range from mosquito vectors carrying malaria and dengue fever to diarrheal disease–related protozoa (e.g., *Cryptosporidium*, *Giardia lamblia*, and *Entamoeba* species) and schistosomes that infect humans contacting water containing infectious cercariae [11]. Food-borne trematodes such as liver, lung, and intestinal flukes also contribute to great morbidity and mortality, largely because of the predilection of many Mekong inhabitants to eat partially cooked or raw fish, some of which are infected with carcinogenic flukes, such as *Opisthorchis viverrini* [12], [13], [14] (Figure 2).

Dam building could potentially trigger an increase in the incidence of many of these neglected tropical diseases, because their epidemiology is inherently linked to wetland ecology and surface water management. For example, dam building may increase the habitat required for the survival and/or reproduction of malaria vectors, such as *Aedes*, *Anopheles*, and *Culex* spp. [11]. Elsewhere, emergences or reemergences of schistosomiasis have resulted from large-scale hydropower projects [15]: e.g., Gezira-Managil Dam (Sudan), Aswan Dam (Egypt), Melkasadi Dam (Ethiopia), and the Danling and Huangshi Dams (China). Similarly, changes in water level and downstream sediment deposition resulting from the building of the Three Gorges Dam in China are expected to increase the schistosomiasis transmission season within the marshlands along the middle and lower reaches of China's Yangtze River [11], [15]. Schistosomiasis naturally occurs in the mainstream Mekong in southern Laos and northeastern Cambodia, and dam building could increase its prevalence.

There is already an example in the Lower Mekong Basin linking infectious disease ecology and anthropogenic changes in surface water. Development projects in Lao PDR aimed at road infrastructure improvement and flood reduction have facilitated the excavation of small aquaculture ponds within villages, which now provide anthropogenic microhabitats in which the full life cycle of *Opisthorchis viverrini* can occur [16]. The threat of an increase in the prevalence of *O. viverrini* further escalates because there are no precautions to prevent the stocking of aquiculture systems with fish that are infected with trematodes, including *O. viverrini*, in nurseries and hatcheries, as was found in the Lao study and another site in northeast Thailand [16], [17].

The linkage between opisthorciasis and home garden ponds in Lao PDR demonstrates the potential for dam building on the Mekong to both directly and indirectly create new habitats where the entire life cycle of *O. viverrini* can be completed. For example, it is likely that aquaculture in small-scale ponds as well as in reservoirs behind dams, will increase to help offset the reduction in wild-captured fish protein caused by dam building—an estimated 340,000 tons, representing the world's most important inland fishery [6]. The threat of the disease magnifies in such confined environments, because free-swimming cercariae have a greater probability of contacting and infecting native fish hosts. Additionally, water released from dams for irrigation and during high-capacity periods can act as vector accumulation sinks, providing new habitats for infected snail and fish intermediate hosts [11].

We recognize a number of uncertainties in our assessment of the linkages between dam building and disease triggers. For example, the risk of disease incidence will probably always vary greatly throughout the Mekong basin. In the case of *O. viverrini*, incidence is in part determined by food-related elements of the culture, namely the predilection of some groups of people to eat insufficiently cooked fish dishes [12]. In areas where infection from aquaculture fish are of concern, introducing exotic fish that are not known hosts of trematode parasites could reduce the risk of increased human infection, but such species might not be accepted in particular types of local dishes because of taste preferences [12], [14].

At a larger scale, the proposed cascade of dams may offset increases in the transition of several types of water-borne and vector-borne communicable diseases that often occurs following large, protracted floods. However, such a positive outcome is uncertain, as floods of this scale are related to unpredictable climatic events, as well as to reservoir storage and release management decisions. With the major focus of Mekong dams being power generation, high water levels are quite likely to be maintained throughout the late part of the monsoon season when tropical storms strike the Mekong basin [18]. Recent floods on the Chao Phraya River in Thailand and the Sesan River in Vietnam were arguably exacerbated by the difficulties of managing reservoirs when catchments were wet, reservoirs were full, and tropical storms struck [18], [19].

Uncertainty aside, political and administrative measures are needed to minimize the impact of disease vectors that benefit from dam building and landscape modifications in the basin. Environmental and social impact assessments should address the negative effects on aquatic biodiversity and food production, as well as changes in infectious disease ecology related to the modification of hydrological patterns and wetland environments. Moreover greater attention should be given to the impacts of dams built on Mekong tributaries, which have received less attention but would also be affected negatively.

While some may believe that building cascades of dams on the Mekong River is an appropriate solution to growing energy demands in the Mekong region, the potential threat to biodiversity, regional food security, food safety, and public health are potentially unmatchable. We believe the long-term impacts likely counter the economic gains of energy export, which will be short-term because of the limited lifetimes of reservoirs (estimated at 50–100 years). More carefully designed dams could reduce or mitigate some impacts, but so far no changes have been proposed to reduce impacts on food safety, disease, and longevity of reservoirs.

An alternative approach for supplying energy locally with fewer negative impacts could involve working with the region's monsoon climate regime, which creates a distinct seasonality with respect to natural resources that could be used for energy generation. Integrated systems could be developed to maximize the generation of solar energy in the relatively cloud-free dry season, pico- or small-scale hydropower in the uplands during the wet season, and wind and geothermal energy in areas and/or seasons that are appropriate. Additionally, green toilet systems could be used for the dual purpose of creating energy and improving the sanitation of human waste that is in part responsible for the presence of some water-borne parasites in wetland systems.
